# Supplementary material for: Identifying common transcriptome signatures of cancer by interpreting deep learning models
Source: Genome Biol. 2022 May 17;23:117. doi: 10.1186/s13059-022-02681-3 (PMC9112525; doi:10.1186/s13059-022-02681-3)
Supplement: Supplementary file 1 — Additional file 1 Supplementary Figs. S1–8. [file 13059_2022_2681_MOESM1_ESM.pdf]

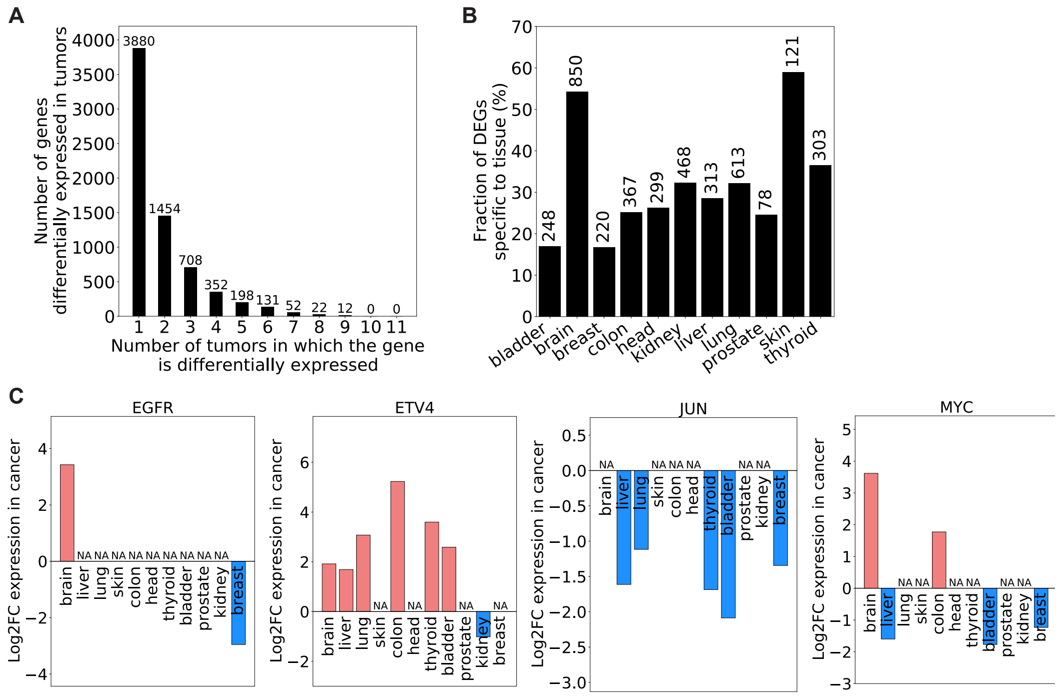

Fig S1: (A) Distribution of tissue specificity for genes that are differentially expression in at least one tumor type. (B) Fraction of differentially expressed genes that are specific to each tumor type. (C) Differential gene expression of four oncogenes in 11 normal tissue-cancer pairs. NA: not differentially expressed.

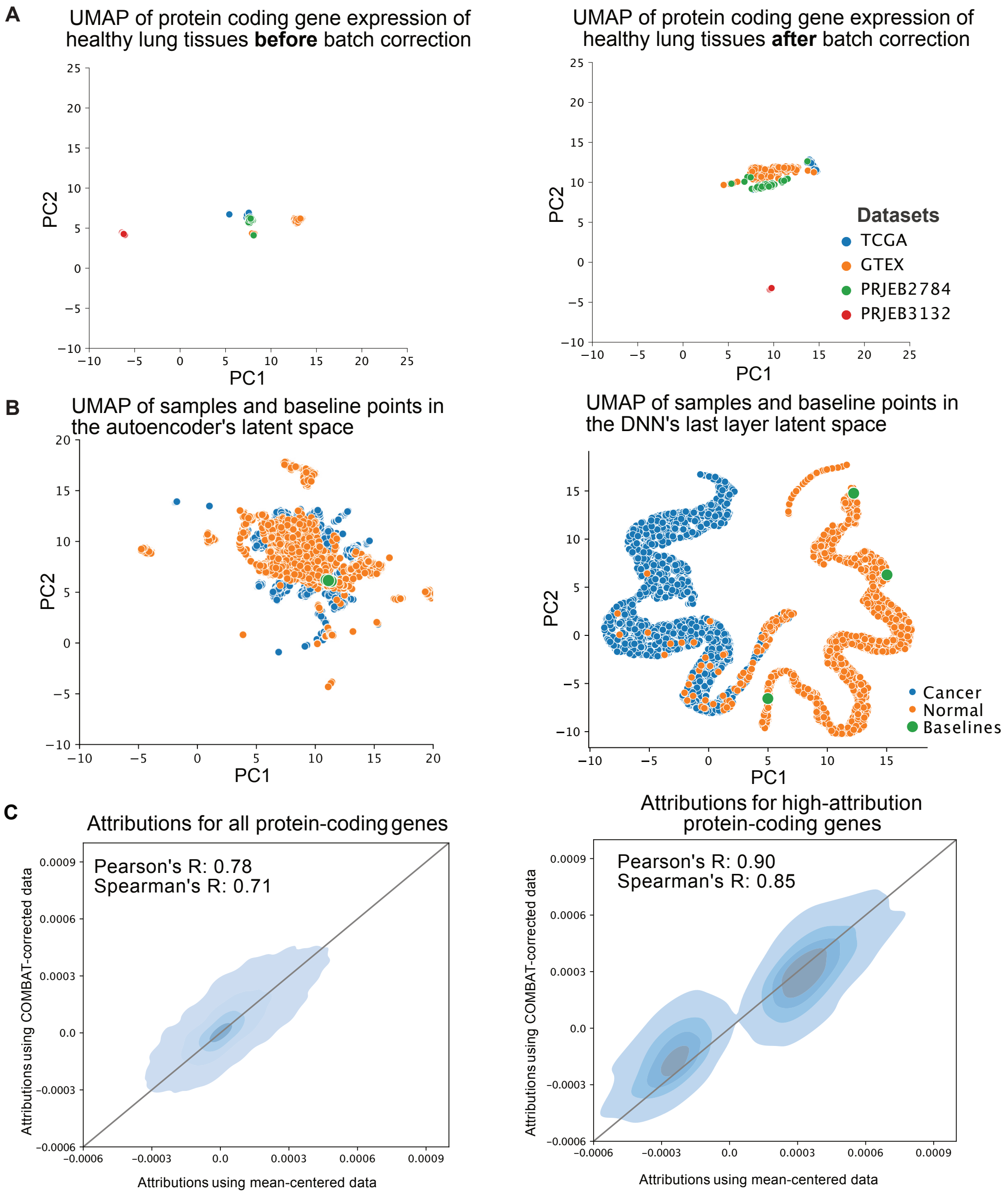

Fig S2: (A) UMAP showing the gene expression of protein coding genes in normal lung samples in four datasets (GTEx, TCGA, PRJEB2784 and PRJEB3132) before (left) and after (right) batch correction. (B) Left: UMAP embedding of the protein-coding autoencoder's latent layer representation of the cancer and normal samples along with the median baseline points from the normal class. Right: UMAP embedding of protein-coding deep neural network's last layer representation of the cancer and normal samples along with the median baseline points from the normal class. (C) Left: Density plot with attribution scores for all protein-coding genes using the deep neural network trained on mean-centered data on x-axis and using the deep neural network trained on COMBAT-corrected data on y-axis. Right: Density plot with attribution scores for 1768 cancer-signature protein-coding genes using the deep neural network trained on mean-centered data on x-axis and using the deep neural network trained on COMBAT-corrected data on y-axis.

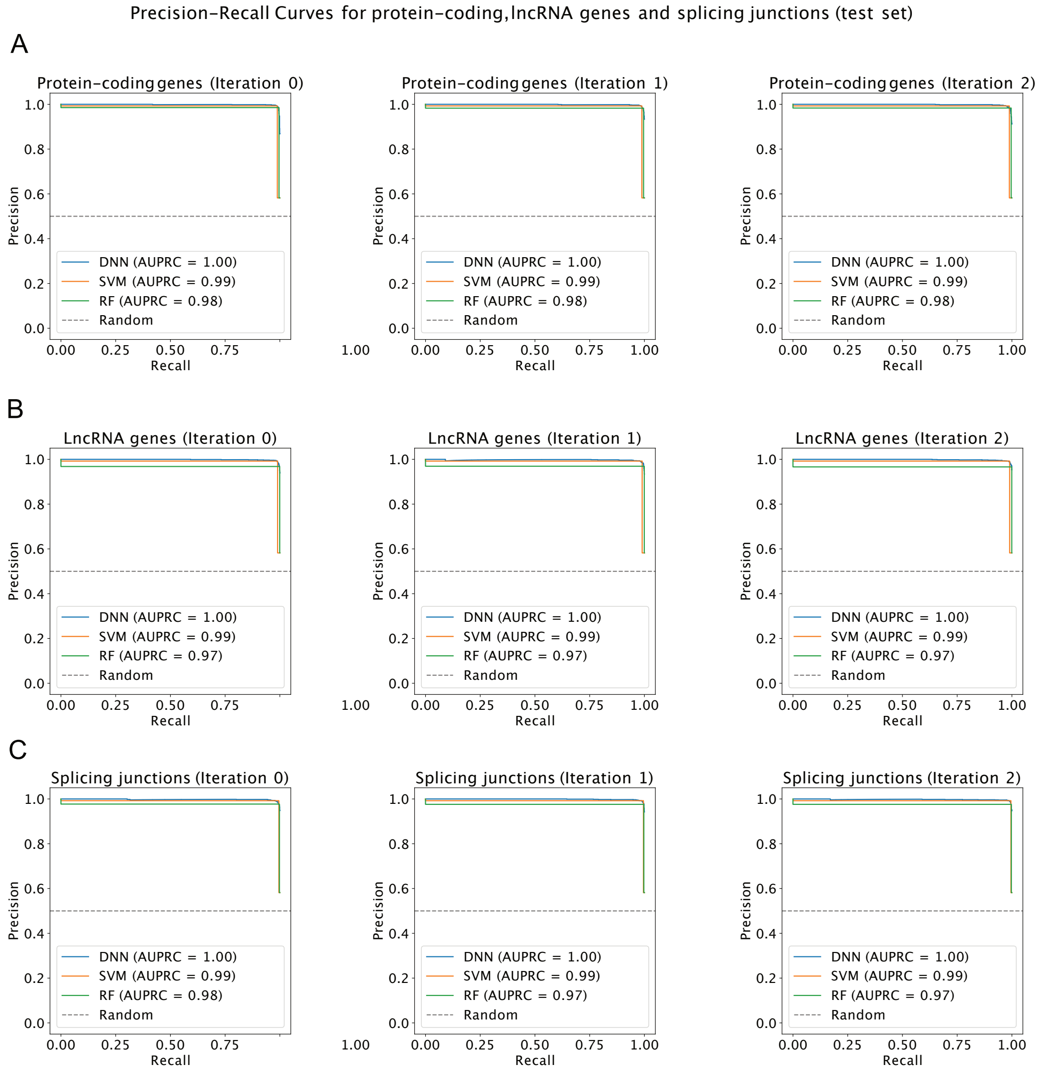

Fig S3: Precision-Recall (PR) curves of deep neural network (DNN), Support Vector Machine (SVM) and Random Forest (RF) models on test set (unseen samples from the tissue/tumor types and datasets present in the training set). (A) Precision-Recall curves for protein-coding genes with DNN, SVM and Random forest models. We repeated the training three different times to compute robust measure of performance for each model along with the standard error. The three PR curves represent each run. (B) Precision-Recall curves for lncRNA genes with DNN, SVM and Random forest models. (C) Precision-Recall curves for splicing junctions with DNN, SVM and Random forest models.

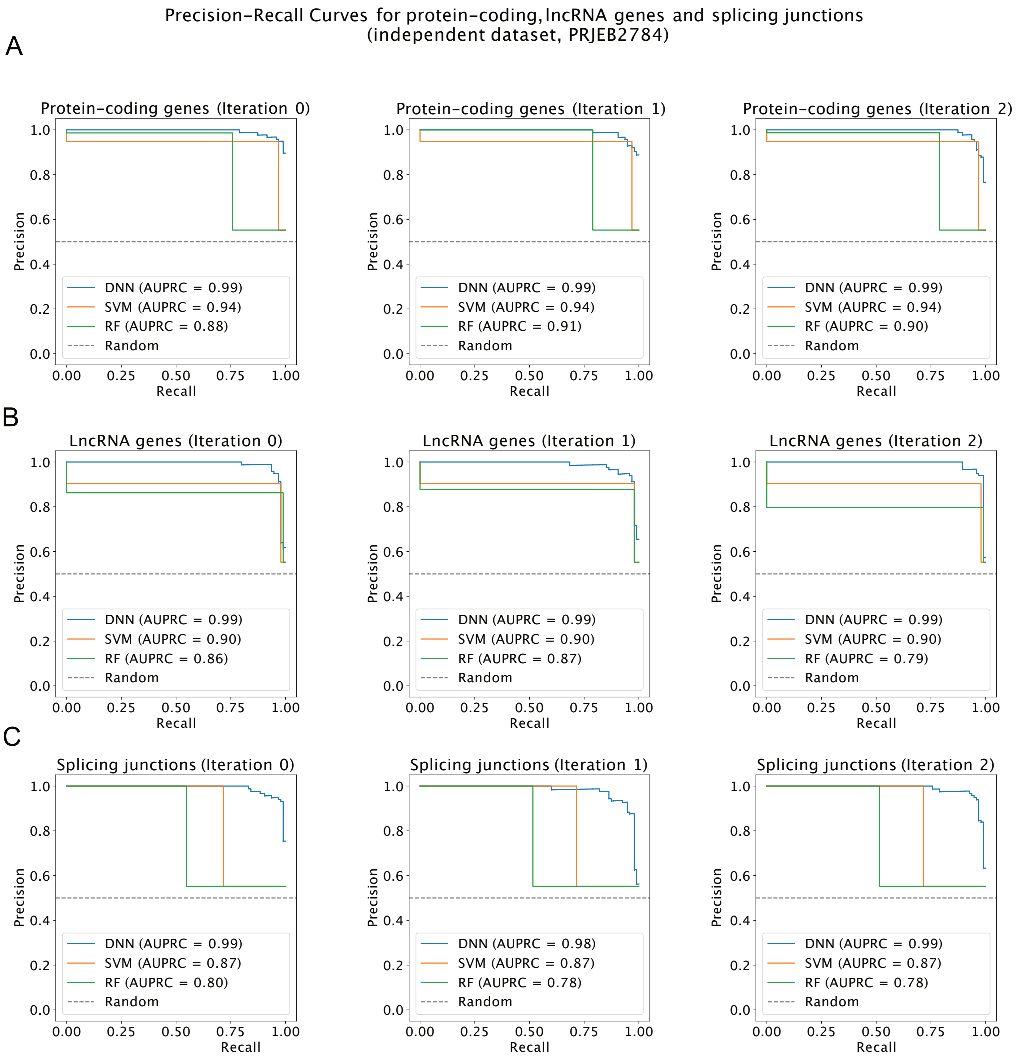

Fig S4: Precision-Recall (PR) curves of deep neural network (DNN), Support Vector Machine (SVM) and Random Forest (RF) models on an independent dataset consisting of normal and cancer lung samples. (A) Precision-Recall curves for protein-coding genes with DNN, SVM and Random forest models. We repeated the training three different times to compute robust measure of performance for each model along with the standard error. The three PR curves represent each run. (B) Precision-Recall curves for lncRNA genes with DNN, SVM and Random forest models. (C) Precision-Recall curves for splicing junctions with DNN, SVM and Random forest models.

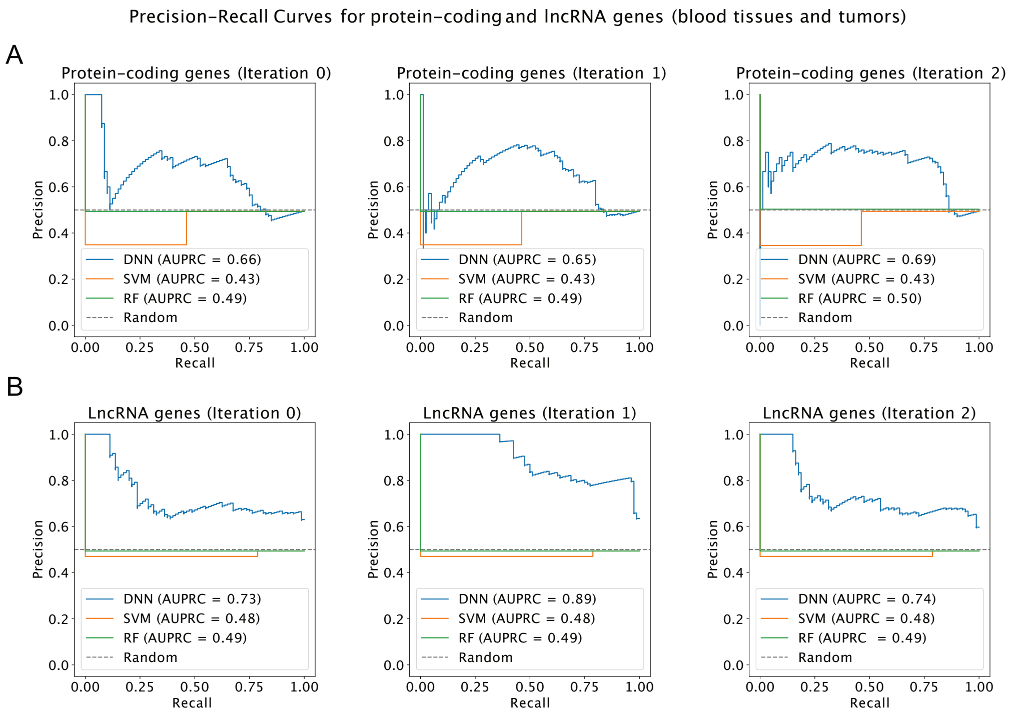

Fig S5: Precision-Recall (PR) curves of deep neural network (DNN), Support Vector Machine (SVM) and Random Forest (RF) models on unseen tissue types with no batch correction and liquid tumors. The training set consists of solid tumors only. (A) Precision-Recall curves for protein-coding genes with DNN, SVM and Random forest models. We repeated the training three different times to compute robust measure of performance for each model along with the standard error. The three PR curves represent each run. (B) Precision-Recall curves for lncRNA genes with DNN, SVM and Random forest models.

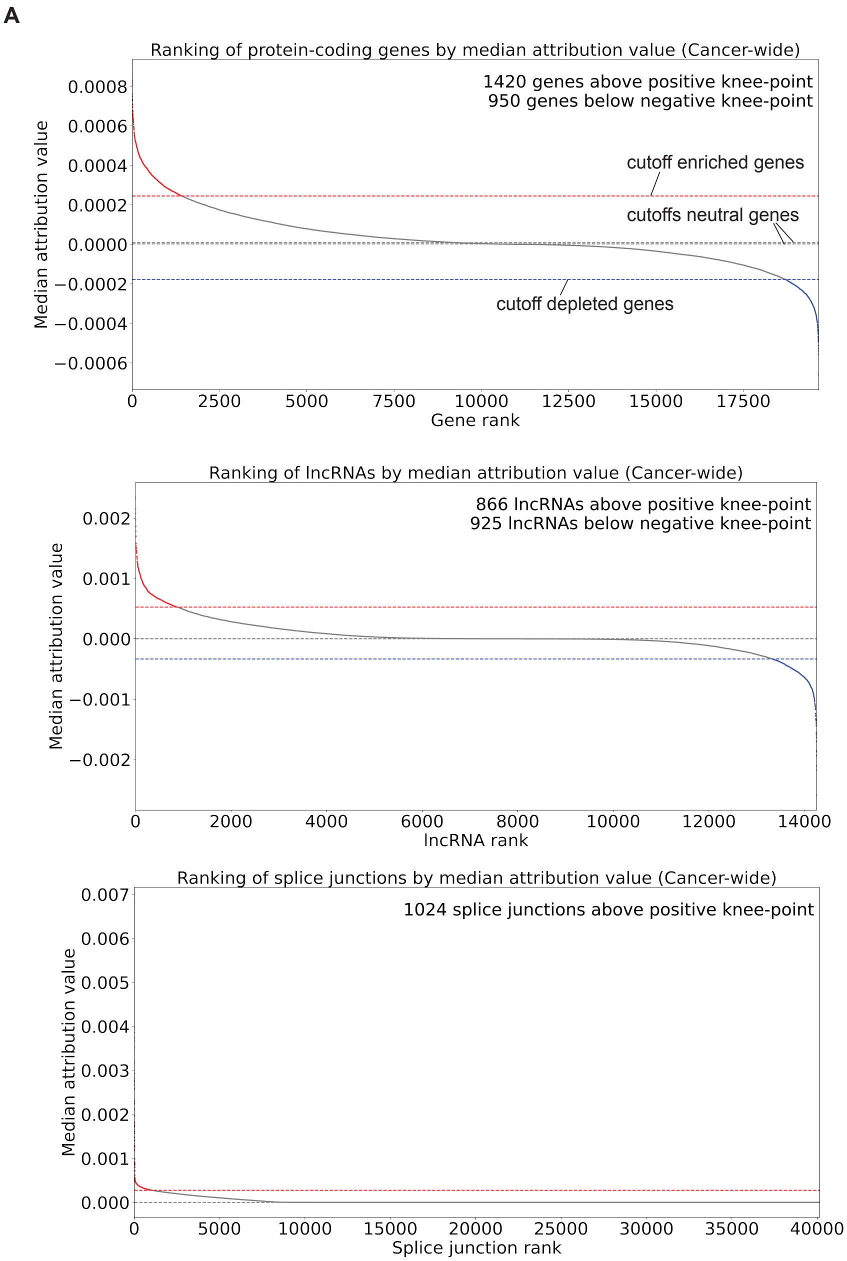

Fig S6: Distribution of transcriptomic features ranked from highest positive values to lowest negative values from models trained on protein-coding gene expression (top), lncRNA expression (middle) or splice junction usage data (bottom). Knee-point of the curves were determined using KneeLocator of the kneed package and are shown with broken horizontal lines.

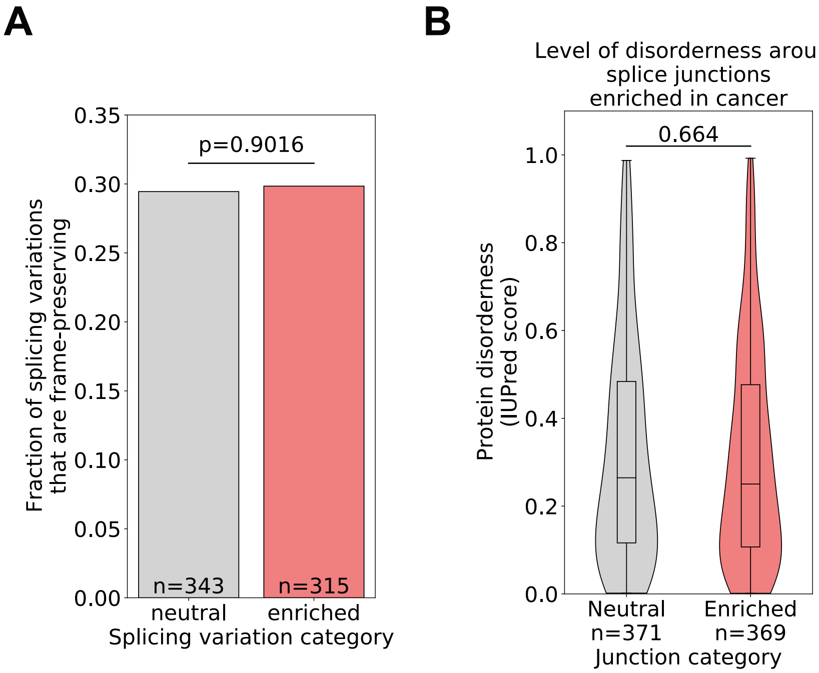

Fig S7: (A) Fraction of splicing variations where reading frame is preserved in both isoforms. (B) Predicted level of peptide disorderness around variable splice junctions.

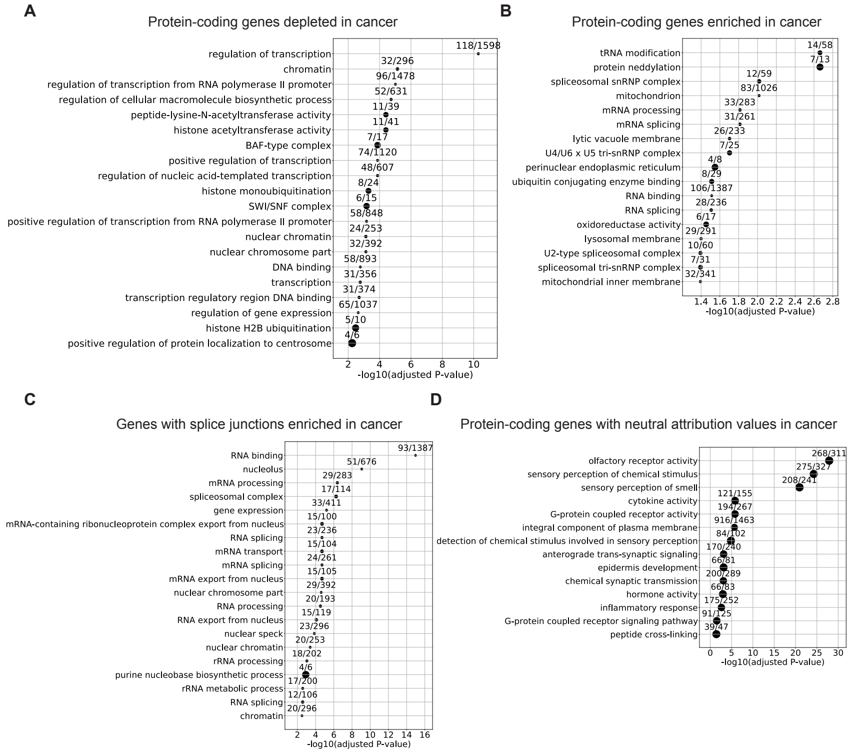

Fig S8: Gene ontology analysis of genes with high negative (A) or high positive (B) attribution values by expression, genes with splice junctions that have high attribution values (C), or genes with neutral attribution values by expression (D). Circle size and values indicate the fraction of genes corresponding to a GO term present in the gene set analyzed. Only top 20 terms are shown if more terms meet our cutoff of adjusted p-value < 0.05.
