# Supplementary material for: Identifying common transcriptome signatures of cancer by interpreting deep learning models
Source: Genome Biol. 2022 May 17;23:117. doi: 10.1186/s13059-022-02681-3 (PMC9112525; doi:10.1186/s13059-022-02681-3)
Supplement: Supplementary file 3 — Additional file 3 Review history. [file 13059_2022_2681_MOESM3_ESM.docx]

Review History

**First round of review**

**Reviewer 1**

**Were you able to assess all statistics in the manuscript, including the appropriateness of statistical tests used?**

No

**Comments to author:**

The manuscript identified common transcriptome signatures of pan-cancers using deep learning models and interpreting them. The motivation of the study is to identify gene signatures commonly shared between cancer types, while predicting tumor and healthy from protein-coding gene expression, lncRNA, or splice junction. The study assessed the method with multiple datasets, mainly including GTEx and TCGA, and explored biological findings by interpreting (using EIG) the deep learning model. The limitation of the traditional analysis (e.g., differential gene expression analysis) brought the motivation of the study well, and biological findings are very well described in a various manner.

Some questions on the paper are below:

1) The proposed deep learning models (simply combination of autoencoder and neural networks) was competitive to the conventional machine learning, including SVM and Random Forest. It was not outstanding performance. What is justification to propose the model?
2) The deep learning model of stacked with autoencoder and neural network may not be innovative. I was wondering the performance of conventional neural network models without autoencoder.
2) In Line 113, the authors concluded that the deep learning model may be robust to unseen data. What may be theoretical justification for that? It may be possible to show better results only the certain data, hematologic dataset?
3) I think that the most important computational part of the paper is to interpret the model using EIG. EIG is a post-hoc interpretation approach, which is independent to models. Then, what would be the justification to use deep learning models? I wondered if the interpretation results may be different from using EIG on SVM or Random Forest, which also showed competitive performance. Then, what are benefits to use deep learning models for the interpretation?

**Reviewer 2**

**Were you able to assess all statistics in the manuscript, including the appropriateness of statistical tests used?**

Yes

**Comments to author:**

The authors proposed using deep learning along with an attribution technique to discover a transcriptional signature of cancer. They collected samples from several data sets and trained three different deep learning models to classify healthy versus tumor samples. The authors provided an extensive set of analyses to interpret the model and understand different aspects of the differences and similarities between the trained models and the characteristics of the highly ranked features. The authors reported the results and described the methods in a highly organized manner and well-written manuscript.

The reviewer, however, raises the following points that may enhance the presentation and solidify the results,

1- It is not clear how the data was split, whether this spit respected the cancer state, the cancer types, or the source dataset, and what ratios were used.

2- The results were reported for one training-testing split. It is not clear if any validation set was used and whether any hyperparameters tuning was done. More importantly, reporting cross-validation results will be helpful in assuring the generalizability of the computational results and the stability of the ranking of the features.

3- Despite the authors reporting that the sign of the attribution score is not informative (not correlated with the biological activity of genes), it is surprising that all the oncogenes are assigned a positive sign while all the tumor suppressor genes are assigned a negative sign in Figure 2C. More justification/discussion will be helpful here.

4- While the authors referred multiple times to the class imbalance, the extent of this class imbalance is not reported clearly in the graphs or the tables. Moreover, the authors didn't report any special handling of the data or the model to solve the problem of the class imbalance.

5- It will be helpful to provide a comparison of the developed model with other reference models using the test set and the independent dataset in terms of the area under the precision-recall curve for the three developed models

6- There is rich literature on automatic features selection in solving the problem of cancer classification. Including this in the introduction will be highly relevant.

7- TCGA and GTEx datasets are highly heterogeneous with several sources of batch effects. It has not been reported that any systemic batch correction tools were used before the analysis. Is the mean centering step enough to deal with the situation?

8- While the main goal of the paper is to find a transcriptional signature of cancer, the identity of the identified signature is not clearly reported in the paper. It may be useful to list the genes involved in this signature. Is this signature computationally useful? For example, can we use this list of genes to accurately identify cancer samples? how many of these genes are known genes? and how many of them harbor genomic alterations?

9- The discussion section can benefit from indicating the potential use cases of such a model and whether there are any clinical applications for the proposed system.

10- The source code is not available to the reviewer

11- Figure 1A The left side of the figure shows the number of differential genes that are unique to each cancer type. However, the support for each cancer type (the total number of differential genes) is not shown and it is not clear how many genes are overlapping with other cancer types. The right side of the graph does not serve a purpose. It only shows that there is no complete overlap between all the cancer types but the extent of the partial overlap is missing from the figure.

12- Figure 1B is confusing a stacked bar chart may be cleaner

13- Figure 1 D is repeated in the caption. Figure parts in the caption need to be renamed to match the figure.

**Authors Response**

**Point-by-point responses to the reviewers’ comments:**

We thank the reviewers for their thoughtful and constructive evaluation of our manuscript. The comments provided by the reviewers allowed us to produce an improved manuscript.

Reviewer #1:
The manuscript identified common transcriptome signatures of pan-cancers using deep learning models and interpreting them. The motivation of the study is to identify gene signatures commonly shared between cancer types, while predicting tumor and healthy from protein-coding gene expression, lncRNA, or splice junction. The study assessed the method with multiple datasets, mainly including GTEx and TCGA, and explored biological findings by interpreting (using EIG) the deep learning model. The limitation of the traditional analysis (e.g., differential gene expression analysis) brought the motivation of the study well, and biological findings are very well described in a various manner.

Some questions on the paper are below:

1) The proposed deep learning models (simply combination of autoencoder and neural networks) was competitive to the conventional machine learning, including SVM and Random Forest. It was not outstanding performance. What is justification to propose the model?

2) The deep learning model of stacked with autoencoder and neural network may not be innovative. I was wondering the performance of conventional neural network models without autoencoder.

*We thank the reviewer for these questions. The justification for the proposed deep learning model are threefold. 1) When compared to SVM and Random Forest, our deep learning model does better (Accuracy, precision, recall) at distinguishing normal versus cancer in previously unseen tissues and when using previously unseen datasets (see Figure 1G, 1H and Supplementary tables 8 and 9). 2) Furthermore, gene expression and splicing measurements are noisy and including an autoencoder allows our model to denoise the data by first learning how to reconstruct these data unsupervised. The latent representation of data thus generated has been shown to perform well on a wide variety of genomic tasks (refs 1, 2, 3, 4, 5 below). An autoencoder also allows us to generate paths for EIG in the latent space of the autoencoder, which we have shown works well for feature attribution (EIG, Jha et al., Genome Biology, 2020). 3) Finally, our focus was on trying to answer a biological question (cancer vs normal) rather than having a perfect model that can predict cancer 100% of the time. We also avoid applying the term “universal” to our models for that reason. Therefore, to keep the focus on interpretation, we tested three different models (SVM, RF and deep learning model) and picked the deep learning model which had the best generalization performance. Given the points raised above about the generalizability of the other models, we also did not find contrasting feature selection by the different methods (which, in turn, would also depend on the selection method and not just the algorithm) to be necessary or particularly informative for the work presented here.


1. Fehrmann, Rudolf SN, et al. "Gene expression analysis identifies global gene dosage sensitivity in cancer." Nature genetics 47.2 (2015): 115-125.
2. Chen, Lujia, et al. "Learning a hierarchical representation of the yeast transcriptomic machinery using an autoencoder model." BMC bioinformatics. Vol. 17. No. 1. BioMed Central, 2016.
3. Tan, Jie, et al. "Unsupervised extraction of stable expression signatures from public compendia with an ensemble of neural networks." Cell systems 5.1 (2017): 63-71.
4. Way, Gregory P., and Casey S. Greene. "Extracting a biologically relevant latent space from cancer transcriptomes with variational autoencoders." PACIFIC SYMPOSIUM ON BIOCOMPUTING 2018: Proceedings of the Pacific Symposium. 2018.
5. Way, Gregory P., et al. "Compressing gene expression data using multiple latent space dimensionalities learns complementary biological representations." Genome biology 21.1 (2020): 1-27.*

3) In Line 113, the authors concluded that the deep learning model may be robust to unseen data. What may be theoretical justification for that? It may be possible to show better results only the certain data, hematologic dataset?

*We thank the reviewer for this question. To show the generalization power of our neural network models, we have done three types of validation in the paper: 1) Performance on unseen samples from tissues/ cancer types seen during training from datasets seen during training (Figure 1E). 2) Performance on unseen samples from tissues/ cancer types seen during training from unseen datasets (Figure 1G and Supplementary table 8). 3) Performance on unseen samples from unseen tissues/ cancer types from unseen datasets (Figure 1H and Supplementary table 9). These validations suggest that our neural network model was able to generalize to new samples in multiple settings including unseen tissues and unseen datasets. All the unseen tissues that we tested are included in the analysis - we did not hand-pick our tissues. We selected blood samples because this is currently a major focus of our other research projects and we had access to extensive high-quality data. However it is entirely possible that we might encounter a tissue/cancer where it would fail if we kept looking. Again, the focus is not to make a perfect, universal model, but one that gives us the biological difference between cancer and normal samples, which we show in our later analyses is what we get.*


4) I think that the most important computational part of the paper is to interpret the model using EIG. EIG is a post-hoc interpretation approach, which is independent to models. Then, what would be the justification to use deep learning models? I wondered if the interpretation results may be different from using EIG on SVM or Random Forest, which also showed competitive performance. Then, what are benefits to use deep learning models for the interpretation?

*We chose to interpret the neural network models because they performed well in all conditions (Figure 1E, 1G) and generalized better than SVM and RF when applied on unseen tissues (Figure 1H and S, Supplementary Table 8B). Furthermore, while it is true that EIG is a post-hoc interpretation method, it can be used only with neural networks and not with SVM and Random forests. The meaning of post-hoc interpretation method in this particular case is that:
1) EIG can be applied to already trained neural networks with no need for retraining.
2) EIG can be applied to different types of neural networks (dense feed-forward networks, convolutional neural networks, recurrent neural networks etc).*

Reviewer #2:
The authors proposed using deep learning along with an attribution technique to discover a transcriptional signature of cancer. They collected samples from several data sets and trained three different deep learning models to classify healthy versus tumor samples. The authors provided an extensive set of analyses to interpret the model and understand different aspects of the differences and similarities between the trained models and the characteristics of the highly ranked features. The authors reported the results and described the methods in a highly organized manner and well-written manuscript.

The reviewer, however, raises the following points that may enhance the presentation and solidify the results,

1) It is not clear how the data was split, whether this spit respected the cancer state, the cancer types, or the source dataset, and what ratios were used.

*We apologize for the lack of clarity in the original manuscript, which we have worked to improve. The samples were split between two classes corresponding to cancer state: normal or tumor. Each class thus comprises several datasets and tissue or tumor types. We added a separate “Class Label” column to Supplementary table 1 which explicitly lists whether each individual sample belongs to the “normal” or the “tumor” class. The distribution of class labels by tissue/tumor types is shown in Figure 1B and distribution by source dataset is shown in Figure 1C. We also clarified the text at lines 80-81: “We assembled a large RNA-Seq dataset comprising 13461 samples from 19 normal tissue types and 18 cancer types and split the data into two classes reflecting cancer state: normal or tumor (Figure 1B; 5,622 total normal samples and 7,839 total tumor samples).” Finally, all references to “normal samples” in the text were changed to “normal samples” to match the term we used to define this class.*

2) The results were reported for one training-testing split. It is not clear if any validation set was used and whether any hyperparameters tuning was done. More importantly, reporting cross-validation results will be helpful in assuring the generalizability of the computational results and the stability of the ranking of the features.

*We apologize that our experimental design was not clearly stated in the text. We divided our dataset into training (8504 samples), validation (2127 samples), and test set (2658 samples). We tuned model hyperparameters (learning rate, hidden layers, number of nodes, activation functions, and dropout probability) on the validation set and fixed our model architecture using the hyperparameters with the best performance on the validation set (see Supplementary Table 3, 5 and 7 for the final model architecture of the DNN model for protein-coding genes, lncRNA and splicing junctions respectively). We then reported the performance of these models on three test sets. First, we evaluated our models on our test set (2658 samples, see figure 1E). Furthermore, we tested our models on test data from an independent dataset consisting of normal and cancer lung samples (Figure 1G and Supplementary Table 8). Finally, we tested the performance of our models on previously unseen tissue types in new datasets without any batch correction (blood cells and blood tumors from another independent source, Figure 1H and Supplementary Table 9). Furthermore, we showed better generalization power of DNN models in comparison to Support vector machine (SVM) and Random forest (RF) on the three previously mentioned test sets. We also showed a high correlation (Pearson’s R: 0.90, Spearman’s R:0.85) between the attribution scores learned by our DNN model with mean-corrected data and the DNN model COMBAT-corrected data (see the response to point 7 for further details). We believe that all these results together suggest that our models have learned a robust transcriptomic cancer signature that can generalize across datasets and tissue/cancer types. We added this information to the text on lines 99 and 138.*

3) Despite the authors reporting that the sign of the attribution score is not informative (not correlated with the biological activity of genes), it is surprising that all the oncogenes are assigned a positive sign while all the tumor suppressor genes are assigned a negative sign in Figure 2C. More justification/discussion will be helpful here.

*This is indeed a very interesting observation that likely reveals an important point about the biological role of high positive or negative attribution genes. Some preliminary investigation of that question revealed that, although the attribution score is not related directly to genes going up or down individually, there was a clear correlation to overall expression level of genes grouped by biological roles (e.g. GO annotation). That said, since we do not have strong experimental support we prefer to err on the side of caution and not overstate our results.

While the network analyses, computational interpretation and experimental validations required to understand the relationship between gene expression level and attribution value extend beyond the scope of this manuscript and will be the object of future research, we did modify the text in the discussion at line 304 to acknowledge the observation shared to by the reviewer:

“In addition, while the directionality of the attribution value does not directly reflect the difference in expression across all cancer types (e.g. a gene with a high positive attribution won’t necessarily have higher expression in all cancer types), we noticed that known oncogenes generally have positive attribution values and known tumor suppressors generally have negative attribution values (Figure 2C). This observation suggests that positive attribution genes could be considered “oncogene-like” while negative attribution genes could be considered “tumor-suppressor-like” in the way they are altered in cancer and, perhaps, in how they contribute to cancer biology.*

*Future work should be directed at assessing whether the transcriptomic features of cancer that we highlight here are causally involved in tumorigenesis or in tumor suppression […]”*

4) While the authors referred multiple times to the class imbalance, the extent of this class imbalance is not reported clearly in the graphs or the tables. Moreover, the authors didn't report any special handling of the data or the model to solve the problem of the class imbalance.

*We thank the reviewer for bringing to our attention the incorrect terminology employed in the text. In referring to “class imbalance” in the text, we actually meant “dataset imbalance” as the classes that are used to train the models (normal vs. tumor) are in fact fairly well balanced (5,622 normal samples, 7,839 tumor samples). Since the models were trained on balanced classes, we did not require special handling of the data to address this. We corrected the text where we referred to “class imbalance” (now “dataset imbalance”) and added the number of samples in each class at line 81.*

5) It will be helpful to provide a comparison of the developed model with other reference models using the test set and the independent dataset in terms of the area under the precision-recall curve for the three developed models
 *We thank the reviewer for this helpful comment. We have added Supplementary Figures S3-S5 with the PR curves and corresponding area under the PR curve (AUPRC). We have updated Figure 1E, 1G, 1H and Supplementary tables 7 and 8 to report AUPRC.*

6) There is rich literature on automatic features selection in solving the problem of cancer classification. Including this in the introduction will be highly relevant.

*We modified the introduction starting at line 45 to acknowledge the wealth of literature existing on the subject of automatic feature selection in cancer classification problems and added several references to publications addressing this problem.*


7) TCGA and GTEx datasets are highly heterogeneous with several sources of batch effects. It has not been reported that any systemic batch correction tools were used before the analysis. Is the mean centering step enough to deal with the situation?

*We thank the reviewer for raising this important issue. In our original submission, we addressed the batch effects in our samples through two means: 1) mean-centering tissues/cancer samples where we had two or more data sources (13/19 normal tissues and 8/18 cancer tissues). 2) showing the generalization power of our deep learning model by evaluating it on two independent datasets (Figure 1G and Supplementary table 8 show the performance of our model on distinguishing normal lung and tumors on a previously unseen dataset. Figure 1H and Supplementary table 9 show the performance of our model on previously unseen blood tissue and tumor on a new dataset without any sort of batch correction).

To address the reviewer’s concern about standard batch correction methods, we ran the commonly used COMBAT (Johnson et al., Biostatistics, 2007) to correct gene expression data for batch effects. First, we note that using COMBAT limits both the data sets and gene sets that can be corrected by its model (e.g., removes ~5.5K genes). There are two specific issues we want to raise here.
a. COMBAT can be applied to individual tissue/cancer types only. This is necessary in order to remove batch effects and preserve biological signals in a tissue/cancer type. Therefore we needed at least two data sources for each tissue/cancer type to apply batch correction using combat properly. We found additional data sources for 13/19 normal tissues and 8/18 cancer tissues (we applied combat to 11/19 normal tissues and 7/18 cancer tissues. The remaining two normal tissues and one cancer type had extreme imbalance, which led combat to filter ~14-16k genes making the corrections unusable). This meant applying combat correction for a subset of tissues/cancers and leaving others unchanged. This would change the underlying data distribution undesirably. For example, TCGA after such a correction would contain both biased and batch-corrected cancer tissues. A machine learning model with this data can, in theory, pick on differences between biased and batch-corrected samples instead of the gene expression signature to distinguish normal versus cancer samples. This is why we opted for mean centering instead of combat and showed the generalization power of our deep learning model by evaluating it on two independent datasets (Figure 1G and Supplementary table 8 show performance of our model on distinguishing normal lung and tumors on a previously unseen dataset. Figure 1H and Supplementary table 9 show the performance of our model on previously unseen blood tissue and tumor on a new dataset without any sort of batch correction).

b. Each of the COMBAT corrections (in total, we applied combat correction to 11/19 normal tissues and 7/18 cancer tissues) filtered out ~9k genes with zero variance in that tissue/cancer type. This criterion filtered out genes with variable expression in other tissues if they had zero variance in the samples being corrected (~5.5k genes fell in this category as only ~3.5k genes had zero variance in all 18 combat runs). In order to include these filtered out genes with variable expression in downstream analysis, we would have to either use their uncorrected gene expression or a zero value. Both these scenarios would change the overall distribution of genes in a sample and potentially introduce spurious signals. Therefore, we opted for mean-centering, which is a simple linear transformation applied to all protein-coding genes instead of a subset of genes.

This original observation, which we did not include in the original submission but now discuss in a paragraph starting at line 86, led us to prefer the mean correction we employed.

Notwithstanding these observations, when we applied COMBAT correction to the tissues/cancer types with two or more data sources, we still found that high-attribution features extracted from a model trained with COMBAT-corrected gene expression data greatly overlap with the high-attribution features we identified with the model trained with mean-centering adjusted gene expression data (see Figure S2C). More generally, we found a very high correlation between the attribution values identified by both types of data pre-processing (see Figure S2C, right panel, Pearson’s R: 0.90, Spearman’s R:0.85). Overall, we believe our new results confirm the validity of our mean-centering approach to address batch effects and highlight the stability of our high-attribution features.*


8) While the main goal of the paper is to find a transcriptional signature of cancer, the identity of the identified signature is not clearly reported in the paper. It may be useful to list the genes involved in this signature. Is this signature computationally useful? For example, can we use this list of genes to accurately identify cancer samples? how many of these genes are known genes? and how many of them harbor genomic alterations?

*We agree that the list of genes composing our transcriptomic signatures should be useful to the community. We now provide lists of high-attribution genes and splice junctions with their corresponding attribution values and p-values in new supplementary tables (Supplementary Tables S10-S12). We also provide the lists of “neutral” features that were used as reference sets in our functional analyses (Supplementary Tables S13-S15).

The fractions of genes composing our signatures that are known cancer genes are reported in Figures 2D and E. The frequency of genomic alterations in high-attribution genes is investigated in Figure 5.*


9) The discussion section can benefit from indicating the potential use cases of such a model and whether there are any clinical applications for the proposed system.

*We now briefly discuss potential clinical application of our signatures at line 299 in the discussion: “Overall, keeping in mind that the models we developed were not designed for clinical application, their robustness across tumor types and the functional properties of their most informative features hint that our signatures could be leveraged to design markers for cancer detection.”*


10) The source code is not available to the reviewer

*We have made all the code publicly available to reproduce the models and analyses on bitbucket: https://bitbucket.org/biociphers/pan-cancer/src/main/*


11) Figure 1A: The left side of the figure shows the number of differential genes that are unique to each cancer type. However, the support for each cancer type (the total number of differential genes) is not shown and it is not clear how many genes are overlapping with other cancer types. The right side of the graph does not serve a purpose. It only shows that there is no complete overlap between all the cancer types but the extent of the partial overlap is missing from the figure.

*We modified Figure 1A to include a bar plot showing the total number of differentially expressed genes for each tissue-cancer pair on the left of the upset plot and changed the right-most part of the upset plot to show the sets that include the most cancer types instead of empty sets. There are too many sets with partial overlaps with only a few genes in each to show all of them in a single plot, but we added a panel in Supplementary Figure 1 that summarizes the number of genes that are consistently differentially expressed in one or more tumor types (Figure S1A) as well as a second panel in Supplementary Figure 1 to show the fraction of uniquely differentially expressed genes for each tumor type (Figure S1B).*


12) Figure 1B is confusing a stacked bar chart may be cleaner

*The use of a stacked bar would be difficult here because we do not systematically have corresponding normal tissue for all tumor types, and vice-versa. In order to make the plot more clear, we grouped tissue types by class, with normal tissues on the left and tumor tissues on the right.*


13) Figure 1 D is repeated in the caption. Figure parts in the caption need to be renamed to match the figure.

*We thank the reviewer for bringing this mistake to our attention. We corrected the legend to Figure 1.*

**Second round of review**

**Reviewer 1**

I appreciate the authors' response. I also checked another reviewer's comments, and it seems the revision reflects most comments of the reviewer. However, I am still not fully convinced that the proposed method is innovative enough to publish in this top-tier journal, since deep learning coupled with autoencoder is a very common approach in this research and the benchmark methods (SVM and RF) did not include current state-of-the-art models. I believe that there are many new proposed methods recently published (past 5 years) for the problem.

**Reviewer 2**

The authors provided satisfactory responses to the issues raised by the reviewer. The manuscript is updated, new supplementary files are provided, and the figures are modified to provide better explanations of the methods, datasets, and the results. One outstanding issue is the that the source code still not accessible using the provided link (<https://bitbucket.org/biociphers/pan-cancer/src/main/>)
